# Ovarian cancer stem cells: still an elusive entity?

**DOI:** 10.1186/s12943-017-0638-3

**Published:** 2017-03-20

**Authors:** Michela Lupia, Ugo Cavallaro

**Affiliations:** Unit of Gynecological Oncology Research, European Institute of Oncology, Via G. Ripamonti 435, I-20141 Milan, Italy

## Abstract

The cancer stem cell (CSC) model proposes that tumor development and progression are fueled and sustained by undifferentiated cancer cells, endowed with self-renewal and tumor-initiating capacity. Ovarian carcinoma, based on its biological features and clinical evolution, appears as a prototypical example of CSC-driven disease. Indeed, ovarian cancer stem cells (OCSC) would account not only for the primary tumor growth, the peritoneal spread and the relapse, but also for the development of chemoresistance, thus having profound implication for the treatment of this deadly disease. In the last decade, an increasing body of experimental evidence has supported the existence of OCSC and their pathogenic role in the disease. Nevertheless, the identification of OCSC and the definition of their phenotypical and functional traits have proven quite challenging, mainly because of the heterogeneity of the disease and of the difficulties in establishing reliable biological models. A deeper understanding of OCSC pathobiology will shed light on the mechanisms that underlie the clinical behaviour of OC. In addition, it will favour the design of innovative treatment regimens that, on one hand, would counteract the resistance to conventional chemotherapy, and, on the other, would aim at the eradication of OC through the elimination of its CSC component.

## Background

### Ovarian cancer

Epithelial ovarian carcinoma (OC) is the most lethal gynaecological neoplasm. Approximately 240,000 new cases of OC are diagnosed every year, with 140,000 patients succumbing to the disease [1]. The 5-year overall survival is below 45% and it decreases to 25% among patients with advanced OC [2]. There are several factors that contribute to the high death-to-incidence ratio of this disease. First, due to the fact that early-stage OC is not associated with specific symptoms, 70% of the cases are diagnosed when the tumor has already spread into the abdominal cavity [3]. Second, even after primary debulking surgery and adjuvant chemotherapy with carboplatin/paclitaxel (see below), the vast majority of patients with advanced OC experience tumor recurrence, in many cases within 2 years from the diagnosis [4]. Third, in contrast to the primary tumor, recurrent disease often develops resistance to conventional chemotherapy, resulting in a very poor cure rate and accounting for the high lethality of OC.

The definition of OC encompasses a wide range of neoplasms that are very distinct for their histopathological traits as well as for their origin, clinical evolution and response to treatment. These different histotypes can be grouped into two main classes: Type I and Type II. The former group, characterized by an indolent clinical course and general confinement to the ovary, includes low-grade and borderline serous, low-grade endometrioid, clear cell, mucinous and transitional (Brenner) carcinomas. These tumors often exhibit mutations in specific genes that include *KRAS*, *BRAF*, *PTEN*, *PIK3CA*, *CTNNB1*, *ARID1A*, and are characterized by microsatellite instability and genomic stability. Type II OC, instead, includes high-grade serous carcinoma, undifferentiated carcinoma and carcinosarcoma, present at advanced stage and exhibit highly aggressive behaviour. These tumors almost invariably have *TP53* mutations, frequent inherited and somatic mutations in *BRCA1* and *BRCA2* genes, and genomic (chromosomal) instability [5, 6]. The most frequent form of type II OC is high-grade serous carcinoma (HGSC), which accounts for about 75% of all OC cases. HGSC is also very aggressive and causes 70–80% of all deaths among OC patients [7], thus representing the most outstanding clinical challenge in gynaecological oncology.

Following primary cytoreduction, patients with Type II tumors undergo adjuvant treatments with platinum-based compounds, often in combination with taxanes. Cyclophosphamide and liposomal doxorubicin are additional chemotherapeutics used in OC treatment. While these drugs have represented the standard of care for the last 40 years (platinum-based therapy was introduced in the late 1970s), other approaches are being intensively investigated especially in combination regimens. For example, the anti-angiogenic agent bevacizumab, an antibody that antagonizes vascular endothelial growth factor, has entered the clinical practice as a first-line therapy in combination to carboplatin/paclitaxel as well as maintenance therapy. Other anti-angiogenic compounds with different mechanisms of action are under clinical investigation [8] and the tyrosine kinase inhibitor cediranib, in particular, prolongs significantly the progression-free survival in platinum-sensitive ovarian cancer [9].

Other therapies that are currently being tested include poly-ADP-ribose polymerase (PARP) inhibitors, which gave promising results in homologous recombination-deficient OC [10, 11], and inhibitors of immune checkpoints (CTLA-4, PD-1, PD-L1) that, however, so far have shown only limited efficacy [12].

## Main text

### Ovarian cancer: biological challenges

As mentioned above, OC defines a number of diseases with different clinical evolution. Such heterogeneity is the result of sharp differences in the biology that underlies the development and the natural history of the OC variants. First, in contrast to the classical view that the different OC hystotypes derive from metaplastic changes of one single tissue, the ovarian surface epithelium (OSE) [13, 14], it has become increasingly clear that only a subset of epithelial OC actually develops within the OSE, while most OC variants originate in non-ovarian districts [15]. As outlined in greater detail below (see “The normal counterpart of OCSC”), this is best exemplified by HGSC, for which clinical, pathological, and experimental evidence supports the fallopian tube as a frequent site of origin [16–19].

OC poses outstanding challenges also with regard to its genomic profile. Indeed, besides the inherent molecular heterogeneity associated with the different tumor histotypes (for example the genomic stability of low-grade serous OC vs. the striking instability of HGSC [3]), the picture is quite fuzzy also within the single variants. Again, HGSC offers a prototypical example in this context: indeed, with the exception of *TP53* that is mutated in virtually all HGSC, there are no mutations in oncogenes or tumor suppressor genes that occur frequently enough to be considered as a hallmark of the disease [20]. Rather, HGSC displays high rate of copy number variations and chromosomal instability which can then result in the inactivation of tumor suppressing pathways as well as in the acquisition of chemoresistance [21].

These and other challenges reflect the difficulties in developing reliable and faithful experimental models of OC. While this has been particularly evident with regard to animal models, as comprehensively reviewed elsewhere [22], recent data have also questioned most of the cell lines that have been used for decades in OC research. Indeed, a number of studies have shown that the majority of classical OC cell lines perform poorly in recapitulating either the molecular pathogenesis and/or the histopathological traits of their supposed tumor of origin [23–26]. Therefore, while these cell lines have provided useful experimental platforms (and are still employed as OC models), the data obtained should be re-interpreted in the light of these re-classification efforts, elucidating in particular to what extent the knowledge acquired can be transferred to the real disease. The studies mentioned above also highlighted a number of OC-derived cell lines that, in spite of their limited use in the past, do recapitulate the genomic profile of their tumor of origin. However, xenograft experiments on some of the cell lines with the highest genomic fidelity highlighted their poor tumor take in recipient mice [27], which may become an issue for their use as preclinical models.

All these challenges related to the OC biology and to its representation should be taken into account when OC models (either in vivo or in vitro) are employed for the identification and characterization of cancer stem cells, and they underscore the requirement for careful validation of the results in clinically relevant settings.

### Cancer stem cells: general concepts

Cancer stem cells (CSC) share several biological features with normal SC, including self-renewal, resistance to apoptosis induced by loss of anchorage, and ability to undergo differentiation through asymmetric cell division. In addition, a defining property restricted to CSC is their ability, upon transplantation into a recipient organism, to generate a tumor in which the hierarchical organization and the heterogeneity of the original disease are recapitulated. Based on this function, CSC are also commonly called tumor-initiating cells [28]. In this review, we will refer to tumor initiation as the ability of a single CSC to form a xenograft representative of the parental tumor. Notwithstanding such a tumorigenic capacity, it should be clarified that the CSC concept is distinct from that of the cell-of-origin. The latter, indeed, refers to the cell from which a tumor has derived, that is the cell type that was first hit by an oncogenic alteration, an event that does not necessarily entails the acquisition of CSC traits [29].

CSC are often resistant to chemotherapeutic and radiation treatments, mainly due to their quiescent state and to the expression of molecular pumps that efflux the drugs and of intracellular scavengers such as ALDH1 (see below). While chemoresistance *per se* is not a defining feature of CSC, it has outstanding implications for the clinical evolution of tumors, for example with regard to recurrence after treatment [30].

Finally, another feature that has been frequently reported in CSC is their acquisition of mesenchymal traits through the so-called epithelial-mesenchymal transition (EMT). EMT was initially characterized as a process that confers migratory and invasive properties to cells [31]. Thereafter, it was found that EMT also endows epithelial cells with stemness-associated properties and, in the case of cancer cells, with increased tumorigenic potential and chemoresistance [32, 33], as it has also been proposed in OC [34]. After the pioneering studies in mammary cells [35], the relationship between EMT and CSC has received further support in several experimental models. In particular, EMT as well as its reversal (mesenchymal-epithelial transition, MET) have been found to be highly dynamic processes in tumor cells, and cancer stemness seems associated with a “partial EMT” phenotype rather than full-blown EMT [33, 36, 37]. This concept is tightly linked with that of CSC plasticity, which postulates that CSC can switch between different states (including non-stem states) [38]. Along this line, partial EMT would represent one of such transitional phenotypes that is compatible with (and may contribute to) the function of CSC.

Biological properties such as self-renewal, asymmetric division, EMT, cancer initiation and differentiation capacity are intrinsically difficult to assess within the original tumor. Therefore, a number of surrogate assays have been developed [39]: for instance, sphere formation under non-adherent conditions reflects the clonogenic potential of CSC (and of normal SC as well) and, upon serial sphere propagation, their self-renewal. Xenotransplantation of immunodeficient recipient mice with a low number of putative CSC allows to determine their tumor initiation ability and their multipotency. While these experimental strategies have obvious limitations and are inherently prone to artifacts, they have been instrumental to elucidate, at least to a certain extent, the complex biology of CSC and their functional implications in a number of tumor types.

Thanks to these research tools, in fact, it has been possible to unravel various signal transduction pathways that play a key role in cancer stemness. These include Wnt/β-catenin, NOTCH, IL6/JAK/STAT3, Hedgehog, NFκB and PI3K/AKT, as outlined in recent and exhaustive reviews [40, 41].

Regarding CSC in ovarian carcinoma (OCSC), other pathways have been found in addition to the ones listed above, and implicated in essential stemness-related processes such as self-renewal, tumor initiation and chemoresistance. These include TLR2-MyD88-NFκB [42], HMGA1 [43], PKCι/Ect2/ERK [44], YAP/TEAD [45], hypoxia/NOTCH1/SOX2 [46] and others that, as discussed below, may also represent useful OCSC markers.

### Cancer stem cells in ovarian cancer: clinical relevance

**Fig. 1:**
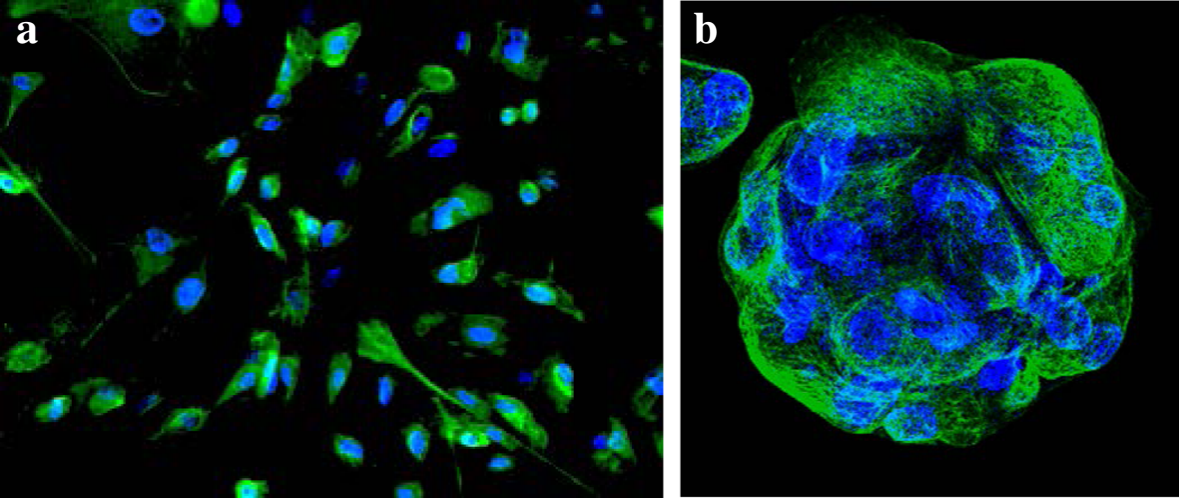
OC-derived sphere. **a** Primary tumor cells were isolated from the ascites of a high-grade serous OC, cultured under adherent conditions, and stained for cytokeratin-8 (CK-8, green), a common marker of OC. Original magnification, 10X. **b** Single cells were then cultured under low-attachment conditions at low cell density and allowed to form clonal spheres. A representative image of a clonal sphere is shown. Sphere-forming cells retained the expression of CK8. Original magnification, 40X

There are many aspects of the biology and clinical evolution of OC which support the hypothesis that this disease is driven and sustained by CSC. For example, OC is often associated with peritoneal ascites where tumor cell spheroids survive and proliferate even in the absence of adhesion to a substrate. The ability to resist *anoikis* (the apoptotic program triggered by the loss of anchorage) is a key property of CSC. In fact, the most widely used in vitro assay for cell stemness, namely sphere formation in suspension cultures (Fig. 1), relies on such a property [47]. Accordingly, ascites is enriched in tumor cells with stem-like properties and has been exploited as a rich source of OCSC [48–54].

CSC frequently exhibit a slow cycling rate which makes them inherently resistant to standard chemotherapy and radiotherapy [55–57] that, by definition, target actively proliferating cells. Thus, the high frequency of OC relapse despite optimal cytoreduction and adjuvant chemotherapy might be accounted for by a subpopulation of quiescent OCSC that survive the treatments. These cells would then “wake up” in a later phase, therefore fueling tumor recurrence. Along the same line, the chemoresistance that develops in most relapsed OC, as opposed to the chemosensitivity found in the majority of primary OC, would depend on the higher frequency of CSC. This hypothesis has received support from various studies: Meng et al., for example, reported the correlation between the relative abundance of OC cells with stem-like traits (CD44^+^/CD24^-^) and the higher likelihood of recurrence as well as shorter progression-free survival [58]. Gao et al. screened a series of paired primary, metastatic and recurrent OC samples for the levels of CD44, a putative CSC marker. A remarkable increase was observed in metastatic and relapsed tumors, toghether with the association with poor outcome. Of note, CD44 was found overexpressed in drug-resistant OC cell lines and up-regulated in mouse models of tumor recurrence following chemotherapy [59]. Similar results were obtained with CD133, another surface molecule frequently associated with CSC, including OCSC: the expression of CD133, indeed, correlated not only with the clinico-pathological parameters of advanced disease, but also with a decreased response rate to chemotherapy and shorter survival [60]. Other studies which have investigated the prognostic power of different OCSC-related markers, such as ALDH1A1 and CD117, have given similar results [61, 62].

Further evidence in support of the clinical implications of OCSC came from functional genomics studies which, based on their gene expression profiles, identified distinct tumor subtypes enriched in stemness-associated genes and associated with poor prognosis [63, 64]. Moreover, several groups have used individual CSC-related biomarkers, including surface antigens, enzymes and transcription factors, to interrogate OC cohorts, and in most cases a correlation with clinico-pathological signs of aggressiveness and/or unfavourable outcome could be established [48, 52, 60, 65–73].

### Phenotype and biology of OCSC: the markers

#### Surface markers

A fraction of cells with clonogenic and self-renewing ability was identified in early studies based on semi-solid supports such as soft agar and methylcellulose [74, 75]. However, the first indication of in vivo validated OCSC came from pioneering studies of Bapat and colleagues who identified cell clones with tumorigenic activity from the ascites of a patient with HGSC, and showed that the tumors obtained in xenotransplanted mice recapitulated the histopathological features of the original disease [48]. Thereafter, several reports described the isolation and characterization of OCSC from patient-derived samples, mouse models of OC, or established OC cell lines. Many of those studies relied on the use of surface markers for the purification of OCSC, as outlined below. Table 1 summarizes some of the information on surface proteins proposed as OCSC markers.

**Table 1:** Cell-surface markers of OCSC

Marker	Biological function	Lowest number of tumorigenic OCSC	References
CD44	HA receptor. Stimulates EGFR-Ras-ERK. Cell proliferation, differentiation, motility, chemoresistance.	10^2^ (CD44^+^/CD117^+^)	[76–85]
CD24	Transmembrane glycoprotein. Activates STAT3. Stemness, cell adhesion, tumor cell malignancy, metastasis.	5x10^3^ (CD24^+^)	[98–103, 105]
CD117	Receptor tyrosine kinase. Regulates PI3K/Akt, Ras/ERK, Src and JAK/STAT pathways. Cell signaling, apoptosis, cell differentiation, proliferation, cell adhesion.	10^3^ (CD117^+^)	[107–112]
CD133	Transmembrane glycoprotein. Induces PI3K/Akt pathway. CSC maintenance, tumor formation, chemoresistance.	10^2^(CD133^+^)	[71, 121–124, 131–136]

See the main text for details and additional references

##### CD44

CD44 has been found associated to the sphere-forming, self-renewing and tumor-initiating fraction of OC cells in different experimental models. Zhang et al. obtained spheroids from primary OC cultures and found sphere-derived cells to be tumorigenic at as few as 10^2^ cells/mouse, while 10^5^ cells were required with the bulk cell population. Spheroids were enriched in CD44-expressing cells, and the authors could reproduce the results obtained with sphere-derived cells by simply xenotransplanting 100 cells with the CD44^+^/CD117^+^ phenotype [76]. Of note, CD44^+^/CD117^+^ cells exhibited also higher resistance to chemotherapeutics [77], thus supporting their CSC-like nature. It is interesting that, in addition to its putative role as OCSC biomarker, CD44 seems also to be involved in OCSC pathobiology, as demonstrated by experiments based on its genetic or functional inactivation [78, 79]. In this context, the molecular mechanisms that underlie the causal role of CD44 in OCSC function could entail its activity as a receptor for hyaluronan (HA), a component of the extracellular matrix. Indeed, the interaction between HA and CD44 triggers a variety of signal transduction pathways. Intriguingly, the binding of HA stimulates the recruitment to CD44 of Nanog, a transcription factor that regulates stemness and chemoresistance in many tumors. The interaction with CD44 induces the activation and the nuclear translocation of Nanog, resulting in the expression of its target genes [80]. The HA-CD44 interaction also promotes the formation of signalling domains at the plasma membrane, which leads to the activation of receptor tyrosine kinase-mediated signalling such as the EGFR-Ras-ERK pathway [81]. This, in turn, may contribute to the proliferation and/or invasiveness of CSC. Finally, CD44 has long been established as a co-receptor for c-Met, the hepatocyte growth factor receptor [82, 83]. While c-Met has been implicated in CSC from different tumor types [84], no information is available on its role in OCSC. On the other hand, c-Met promotes ovarian cancer progression [85]. On these premises, it is conceivable that the CD44/c-Met signalling unit plays a role in OCSC function, thus representing a potential therapeutic target.

The screening of OC sample cohorts lent further support to the association of CD44 with stemness traits and with the clinical course of the disease. As mentioned above, recurrent OC expressed higher levels of CD44 as compared to matched primary tumors [59], and CD44 levels correlated with poor outcome in several studies [73, 86, 87], including a recent meta-analysis on 957 cases [72]. The latter study, however, reported no association with the response to chemotherapy, which is somehow counterintuitive for a CSC marker. In addition, a number of studies either found no correlation of CD44 with patients’ survival [88, 89] or even reported CD44 to be associated with better outcome [90–92]. While there might be several reasons that account for such a discrepancy, including different experimental approaches and molecular tools (e.g., CD44 antibodies), different inclusion criteria between the patient cohorts and different degrees of tumor heterogeneity within the individual cohorts, it appears that the clinical value of CD44 as an OCSC biomarker remains controversial. A possible solution would be to focus on alternatively spliced variants of this molecule. Indeed, CD44 exists in various isoforms, depending on ten exons that can be added in different combinations to the standard form of the molecule [93]. While the functional and clinical implications of CD44 variants have been investigated in various tumor types, only limited information is available on their role in CSC [94, 95]. In OC, in particular, CD44v6 was recently shown to be up-regulated in peritoneal metastasis and, more important, a fraction of CD44v6^+^ tumor cells displayed metastasis-initiating activity [96], pointing to this variant as a putative marker of OCSC.

##### CD24

The heat-stable antigen CD24, a glycosylphosphatidylinositol-anchored membrane glycoprotein, has been extensively used as a negative or positive marker of CSC in various cancer types. For example, the low or absent expression of CD24, in combination with high CD44, marks breast cancer stem cells [97]. Data obtained in various laboratories are consistent with the role of CD24 as a positive marker of CSC in OC. Using a mouse model of OC based on the tissue-specific deletion of *Trp53*, *Pten* and *Apc*, Burgos-Ojeda et al. identified CD24^+^ cells as the tumor-initiating cells. Gao et al. isolated the subset of CD24^+^ cells from OC specimens and demonstrated that this cell subpopulation displayed higher expression of stemness-associated genes and, more important, was endowed with high tumor-initiating potential [98]. Li et al. supported these observations with experiments on established OC cell lines, showing not only that the expression of CD24 increased in sphere-forming cells, but also that neutralizing CD24 interferes with their ability to overcome *anoikis* and generate spheres [99]. Accordingly, tumor cells in OC peritoneal effusions were reported to express higher levels of CD24 than solid tumors, which was proposed as a sign of enrichment in CSC traits [100].

While the functional role of CD24 in OCSC (and in CSC in general) remains poorly defined, recent data have provided novel insights. In particular, CD24 has been shown to enhance the activation of signal transducer and activator of transcription 3 (STAT3) in different tumor types [101–103]. STAT3 activity is a well-established player in cancer stemness and, indeed, CD24-induced STAT3 activation in nasopharyngeal carcinoma cells triggers their reprogramming towards a CSC state [103]. While the causal role of the STAT3 pathway in OCSC has been described [104], its regulation by CD24 remains to be explored. In agreement with the hypothesis of a CD24/STAT3 interplay in ovarian cancer stemness, CD24^+^ OCSC exhibit increased levels of STAT3 phosphorylation and of STAT3-dependent expression of stemness factors such as Nanog and c-Myc [105].

It should be mentioned, however, that there are studies which found no evidence for the association of CD24 expression in OC cells with stem cell phenotype or chemoresistance [68]. In fact, other groups including ours (Lupia and Cavallaro, unpublished observation) attributed CSC properties to subsets of OC cells with no or low expression of CD24 [58, 106].

##### CD117

The *c-KIT* proto-oncogene codes for a receptor tyrosine kinase called CD117, which acts as the receptor for stem cell factor. The role of CD117 as a marker of OCSC has been proposed by several groups. Higher expression of CD117 was described in OC-derived spheres as compared to their adherent counterpart, both in fresh tumor tissue [107] and in OC cell lines [108]. The side population (i.e., a putative stem cell subset; see the section “Side population” below) of a cell line derived from a transgenic mouse model of HGSC was also found enriched in CD117-positive cells, while this was not the case for a cell line established from a mouse model of endometrioid OC [109]. This might imply that different OC subtypes express a different repertoire of surface CSC markers.

Furthermore, as mentioned above, the combined expression of CD117 and CD44 defined a subpopulation of OC cells endowed with tumor-initiating capacity and chemoresistance [76, 77]. CD117 was also efficient in the isolation of OCSC as a single marker: Luo et al., indeed, showed that the <2% fraction of CD117^+^ cells isolated from OC xenografts had a tumorigenic potential 100-fold higher than CD117^-^ cells. Tumors derived from CD117^+^ cells, in addition, recapitulated the heterogeneity of the original disease and could be serially transplanted, confirming the differentiation and self-renewal abilities of these cells [110]. In other experimental systems, however, CD117^+^ OC cells failed to show increased tumor initiation with respect to CD117^-^ cells [105].

The receptor tyrosine kinase activity of CD117 regulates a wide spectrum of signalling cascades, including PI3K/Akt, Ras/ERK, Src and JAK/STAT [111], all of which are plausible candidates as functional effectors of this surface protein in OCSC. Unfortunately, despite the numerous studies that capitalized on CD117 as an OCSC marker, very little knowledge is available on its biological role in these cells. Functional experiments conducted in two OC cell lines revealed that both gene silencing and pharmacological inhibition of CD117 kinase activity with imatinib reduced significantly their sphere-forming potential [112], pointing to CD117 as a possible therapeutic target in the context of OCSC. The same study implicated the Wnt/β-catenin signalling cascade as an effector of CD117 in the regulation of stemness function [112].

Several multi-kinase inhibitors which have CD117 among their targets have been investigated as therapeutics in ovarian cancer [113–115]. Nevertheless, none of these studies has studied whether such drugs affect the OCSC subpopulation. Future efforts should be devoted to understanding the biological role of CD117 in OCSC and to testing CD117-targeted compounds as OCSC-directed therapies.

##### CD133

After the initial characterization as a CSC marker in glioblastoma [116, 117], the surface protein CD133 (also known as prominin-1) was utilized in the identification and/or isolation of CSC in a wide spectrum of solid tumors. However, while harnessing CD133 has allowed significant progress in our understanding of CSC biology, a number of limitations and conflicting data have emerged calling for more caution in the use of CD133 as a widespread CSC marker [118, 119]. OC has not been an exception in this regard. A recent meta-analysis of over 1000 cases has shown a significant correlation between CD133 expression and shorter survival and tumor stage, while no correlation was found with tumor grade or response to therapy [120]. Various groups have detected CD133 in OC-derived subpopulation of cells endowed with properties of tumor initiation, self-renewal and/or chemoresistance [71, 121–124]. Interestingly, the expression of CD133 was silenced by epigenetic mechanisms in the CD133^-^ progeny of CD133^+^ OCSC [125], implicating chromatin modifications in the switch towards a more committed state. Nevertheless, a number of studies did not support the association of CD133 with OCSC. For example, OCSC identified in two cell lines through the expression of ALDH1 (as discussed below) showed inconsistent expression of CD133 [106]. More important, CD133^+^ cells isolated from primary OC cultures failed to prove more spherogenic or tumorigenic than their CD133^-^ counterpart, and in fact they only displayed a slower proliferation rate [126, 127]. Some of the data published point to the phenotypic heterogeneity and/or plasticity of OCSC with regard to the expression of CD133 [67, 128], which may, at least in part, account for the controversial findings discussed above. In this context, also the different pattern of immunoreactivity of different anti-CD133 antibodies [129] could underlie discrepancies in the identification of OCSC. The inconsistent data on CD133 as a surface marker in OCSC could also be accounted for by its dynamic subcellular localization, as shown in other tumor types. For example, cytosolic CD133 is a hallmark of highly aggressive gastric cancer [130] and CD133 was found to interconvert between cytosolic and plasma membrane localization in glioblastoma stem cells [131]. Finally, CD133 has been proposed to mark a subset of cells that contribute to OC vascularization, either by entering an endothelial differentiation program [126] or through a paracrine, pro-angiogenic function [132].

The functional contribution of CD133 to the pathophysiology of CSC, including OCSC, is poorly defined. Nevertheless, various lines of evidence suggest that CD133 is not only a surface marker but it also plays a causal role in CSC. For example, CD133 is required for the maintenance of glioblastoma stem cells [131] and confers stem-like properties and chemoresistance to other cancer cell types [133–135]. Experimental data implicated the PI3K/Akt pathway as a CD133 effector [133, 134, 136]. Akt pathway inhibitors, are being actively investigated in ovarian cancer therapy [137]. It would be worthwhile, therefore, to study the involvement of Akt downstream of CD133 in ovarian cancer and, in particular, to assess whether targeting Akt signalling affects the self-renewal and tumorigenic potential of CD133-positive OCSC.

#### Functional markers

The definition of “functional markers” refers to stemness-associated biological activities or functional states that have been exploited for the prospective isolation of OCSC.

##### ALDH1

Aldehyde dehydrogenases (ALDH) are enzymes that promote the oxidation of aldehyde substrates to their corresponding carboxylic acids. Within the ALDH family, the ALDH1 subgroup is particularly active in normal and cancer stem cells. In particular, among the ALDH1 isozymes (ALDH1A1, ALDH1A2 and ALDH1A3), ALDH1A1 is prominently expressed in CSC. Therefore, ALDH activity and ALDH1A1 expression have been exploited for the identification and purification of CSC across many different cancer types [138]. While the specific role of ALDH1A1 in CSC has not been completely elucidated yet, the protective function of its detoxifying machineries against different insults (preventing for example the accumulation of reactive oxygen species and of reactive aldehydes) is certainly involved in CSC maintenance. In this context, ALDH1 also confers resistance to chemotherapeutics and to radiation [138]. Notably, the widespread use of ALDH1 activity as a CSC marker is largely due to the possibility of determining this activity in live cells and of isolating ALDH1-positive cells with a fluorescence-based assay (Aldefluor, StemCell Technologies, Durham, NC, USA). Direct evidence that ALDH1 activity defines a subpopulation of OC cells with CSC-like properties was provided in numerous studies [53, 61, 67, 106, 127, 139]. In particular, a recent report established the superiority of ALDH1 over CD133 in identifying primary OC-derived cells expressing stemness genes and capable of self-renewal and tumor initiation [127]. Along the same line, the ALDH^+^/CD133^+^ fraction of OC primary cells was identified at the apex of an OC hierarchy and showed a more multipotent phenotype than all the other marker combinations, including ALDH^-^/CD133^+^ [123]. Data from Condello et al. suggested that ALDH1A1 in OCSC is regulated at the transcriptional level by the Wnt/β-catenin pathway, and revealed that a small-molecule ALDH1A1 inhibitor abolished sphere formation, pointing to this enzyme as a potential therapeutic target [140]. Of note, knockdown of the *ALDH1A1* gene in OC cell lines restored their sensitivity to chemotherapy both in vitro [141] and in mouse xenograft models [61].

While some of the studies discussed above pointed to the causal role of ALDH1 in conferring CSC traits to OC cells, the underlying molecular mechanism have not been fully elucidated yet. Recent data suggested that ALDH1A1 exerts a regulatory function on the levels of ATP-Binding Cassette (ABC) drug transporters, thus modulating the resistance of OC cells to chemotherapeutics, although the molecular mechanisms remains to be pinpointed [141]. Interestingly, the role of ALDH1 in OCSC extends beyond its detoxifying activity: Meng et al. showed that the knockdown of ALDH1A1 in the OC cell line A2780 caused a decrease in the cell cycle checkpoints regulators KLF4 and p21 which, in turn, resulted in enhanced cell proliferation [142]. Actively proliferating cells are more susceptible to cytotoxic drugs and, therefore, forcing the cell cycle entry likely contributes to the sensitization of OC cells to chemotherapy upon loss of ALDH1A1. Furthermore, the ablation of ALDH1A1 triggered DNA damage with a concomitant reduction in various DNA repair pathways [142], implying that ALDH1A1 exerts a genome-protecting role in OCSC. The anti-proliferative role of ALDH1A1 reported by Meng et al. was contradicted by data obtained in primary OC-derived cells where the genetic or pharmacological inactivation resulted in decreased proliferation [127]. In this experimental system, the authors also identified an intriguing interplay between ALDH1A1 and the stemness-associated gene *SOX2* and showed that their reciprocal regulation orchestrates sphere formation and OCSC survival and proliferation [127].

##### Side population

Besides ALDH1 activity, the functional feature that has been most extensively used for the isolation of OCSC is the ability to efflux the lipophilic dye Hoechst 33342 due to the selective expression of ABC transporters. Cells endowed with such a property are called side-population (SP) cells due to their position in FACS panels [143]. In OC, this method was first applied to the identification of tumor-initiating cells from the transgenic mouse model of OC known as MISIIR-TAg [109]. Thereafter, increased expression of stemness-related genes and tumorigenic capacity were reported in SP cells isolated from a panel of OC cell lines as well as from human tumors [144, 145]. Of note, the SP fraction purified from different OC cell lines was highly heterogeneous with regard to the expression of other markers [146], raising the question of whether cells with multiple phenotypes can co-exist in the CSC compartment or one single subset contained in SP cells represents *bona fide* OCSC.

##### Label retention

**Fig. 2:**
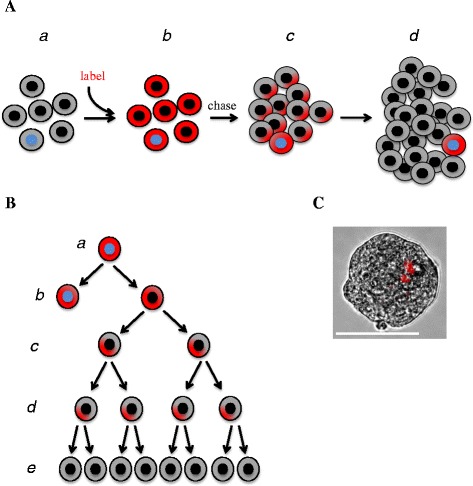
Label retention in CSC. ***(A)*** Schematic representation of label retention. A bulk population of tumor cells (*a*) is labeled with a vital dye so that all cells become labeled (*b*). During the following chase period, actively dividing cells (black nuclei) progressively lose the dye by diluting it to daughter cells (*c,d*). In contrast, CSC (blue nucleus), due to their slow cycling rate, retain the dye much longer and can thus be identified (*d*). ***(B)*** Label retention during sphere formation. When labeled, single CSC (*a*) are cultured under low-attachment conditions they undergo asymmetric division to generate another CSC and a progenitor cell (*b*). While the daughter CSC enters quiescence thus retaining the dye (*b*), the progenitor give rise to a progeny of proliferating cells (*c*), which form the bulk of sphere cells, in which the dye is progressively lost (*d,e*). The final result, as shown in ***(C)***, is a clonal sphere with only one or very few label-retaining CSC. The image shows a sphere from primary OC cells labeled with PKH26. Scale bar, 100 μm

In various tumor types, CSC are thought to be mostly quiescent or at least to have a very slow cycling rate. This property has been harnessed by a number of laboratories to isolate quiescent CSC from the bulk of actively proliferating tumor cells. In particular, this type of technology is based on the principle, schematically depicted in Fig. 2, that, upon labelling a cell population with a fluorescent vital dye, the latter will be progressively diluted in dividing cells while quiescent cells will retain it and can be purified by FACS-based strategies. For example, retention of the lipophilic dyes of the PKH class (which intercalate in the cell membranes) was used for the isolation of quiescent OCSC and allowed to demonstrate the reversibility of their state to an active proliferating phenotype when their clonogenic or tumorigenic function is stimulated [147]. Recently, by applying the PKH technology to an in vivo model of OC, Bapat et al. identified gene modules specifically associated to the individual tumor cell fractions separated on the basis of their PKH retention, and defined CD53 as a novel marker of OC-initiating cells within the PKH^high^ subset [148]. PKH retention was also used as a proof of quiescence during the characterization of polyploid giant cancer cells as OCSC [149].

The isolation of label-retaining OCSC entails, by definition, the manipulation of the system (e.g., biochemical or genetic labelling, expansion of tumor cells to allow for label dilution, etc.), which obviously increases the risk of artifacts. In addition, label retention marks quiescent cells which, however, not necessarily coincide with CSC. These limitations should be taken into account when applying label retention-based strategies. Yet, the latter offer the possibility to discover molecular and biological features of CSC, provided that the findings are validated in clinically relevant systems. For example, the transcriptomic profile of PKH-retaining mammary cells has yielded a panel of differentially expressed genes that have then been proven useful for the prospective identification of CSC from breast tumor tissue [150]. Quiescent CSC can also be tracked genetically, for example through the retention of green fluorescent protein-tagged histone 2B (H2B-GFP). This approach can be combined with animal models of spontaneous tumorigenesis, thus making it possible to identify label-retaining CSC in preclinical tumor models that recapitulate, at least to some extent, the natural history of the disease. Long-term retention of H2B-GFP favoured the identification and characterization of stem cells in the ovarian and oviductal epithelium of transgenic mice [151, 152]. Applying this strategy to mouse models of spontaneous ovarian carcinogenesis should help defining novel molecular and functional features of OCSC, not only in the context of tumor initiation, but also in dissemination, recurrence and chemoresistance.

It should not be neglected, however, that PKH-retaining cells isolated from OC specimens failed to show higher tumorigenic potential when compared to their PKH-negative counterparts and, in fact, exhibited lower clonogenic activity in vitro and longer tumor latency in xenograft models [153]. Results in our laboratory with either PKH or H2B-GFP indicated that label retention in primary OC cells does not mark cells with specific OCSC-like traits (Lupia and Cavallaro, unpublished data). These observations might imply that OC is highly heterogeneous with regard to the quiescent nature of its CSC compartment, and that slow cycling is not an absolute and universal feature of OCSC.

### The normal counterpart of OCSC

The origin of CSC remains a highly debated and controversial issue. Based on the functional and phenotypic similarities between CSC and normal tissue stem cells (self-renewal, differentiation, quiescence, shared markers, etc.), CSC were initially proposed to derive from normal SC that have undergone malignant transformation. More recent evidence, however, pointed to the dedifferentiation and reprogramming of “mature” tumor cells as an additional mechanism to generate *bona fide* CSC. Furthermore, it has been proposed that tumor cells convert dynamically between stem and non-stem states, a plasticity that can be orchestrated by microenvironmental cues [38]. These models are not mutually exclusive, and it is likely that different pathways underlie the origin of CSC in different tumor types. In addition, different mechanisms may occur within the same tumor [38], possibly accounting for the generation of distinct CSC subsets. The intra-tumor heterogeneity of CSC, indeed, has been reported in certain cancer types, one of the best examples being ovarian cancer [146, 154].

Regardless whether CSC originate from normal stem cells (SC) of the same tissue, investigating the latter has provided significant insights into the biological features of CSC. For example, certain signalling pathways governing the stem phenotype in CSC have been identified also in normal SC, and in some cases signature inferred from normal SC have been used to prospectively identify the CSC in the corresponding tumor tissue [30, 150]. In addition, comparing normal SC with CSC might help to identify molecular drivers that act specifically in CSC, possibly resulting in a better understanding of CSC biology and in CSC-targeted therapies.

In the context of OC, this approach must face both biological and methodological challenges mainly linked to the tissue of origin and, therefore, to the somatic SC that one should consider as the normal counterpart of OCSC.

OC has long been thought to derive from the neoplastic transformation of cells belonging to the ovarian surface epithelium (OSE), a monolayer of cells with mesothelial characteristics lining the ovary [13, 14]. Recent clinical and experimental data, however, have demonstrated that a significant fraction of OC originate in non-ovarian tissues [155]. This is best exemplified by HGSC, for which the precursor lesion is often localized within the distal fallopian tube epithelium (FTE), and is driven by *TP53*-mutated FTE cells [17, 18]. A recent phylogenetic analysis of the tumor mutational profile has confirmed the frequent tubal histogenesis of HGSC, although in some cases the FT itself appeared to be a metastatic site [156].

From a methodological standpoint, the poor accessibility and the difficult handling of OSE and FTE have posed major obstacles to the identification and characterization of SC residing in these tissues. Nevertheless, as discussed below, a few studies have addressed this question and provided intriguing information.

#### OSE stem cells

Tracing quiescent cells through their long-term retention of 5-bromo-2-deoxyuridine (BrdU) and H2B-GFP has revealed a subset of SC-like cells in OSE of adult mice which displayed asymmetric cell division and higher clonogenic potential when compared to their non label-retaining counterpart [151]. The SP analysis (described above) was instead employed by Gamwell et al. [157] to isolate rare SC from mouse OSE which expressed the classical SC marker Ly6A/Sca-1 and had higher sphere-forming efficiency. These studies, however, did not explore the possible relation of OSE-derived SC with OCSC. Such a question, instead, was address by the researchers who identified a SC subpopulation in the ovary hilum, which is a transitional area between the OSE, mesothelium and the oviduct. Besides exhibiting a wide spectrum of phenotypical and functional features classically associated with stemness, hilum-derived SC were more susceptible to the neoplastic transformation induced by the deletion of *Trp53* and *Rb1*, implying that the ovary hilum may act as the niche for OCSC [158]. This study showed that OSE-derived SC express the surface protein Lgr5, previously characterized as a SC marker in different epithelia [158]. Subsequent in vivo tracing identified Lgr5^+^ SC not only in the hilum but also in other regions of the OSE and in the tubal epithelium [159]. Of note, this paper documented the role of Lgr5^+^ cells in OSE homeostasis and in epithelial regeneration following ovulation-induced damage, providing compelling evidence of their SC nature [159].

#### FTE stem cells

In addition to Ng et al. [159], a few other studies have addressed the identification of SC in the fallopian tube. Pulse-chase experiments in mice expressing H2B-GFP in an inducible fashion revealed the presence of long-term, label-retaining (i.e., quiescent) cells in the distal portion of mouse oviduct. These cells were capable of sphere formation of differentiation towards different lineages of the female reproductive system [152]. Label-retaining cells in mouse oviduct were also identified through pulse-chase experiments with BrdU, although their SC nature was not further investigated [160]. Paik et al. [161] identified a subpopulation of basally located cells that do not express the typical markers of ciliated or secretory cells, the two cell lineages that compose the FTE, while expressing CD44. These cells, termed FTESC and found to correspond to the so-called peg cells, were enriched for sphere-forming activity. Notably, an expansion of FTESC was found both in HGSC lesions and in the normal appearing FTE adjacent to HGSC sites [161], possibly implicating FTESC in the pathogenesis of this cancer type. Direct functional evidence in support of the causal link between human FTE-derived SC and OC development was provided by the in vitro immortalization with *hTERT* and transformation with *c-MYC*, an oncogene that is frequently overexpressed in OC. Besides having tumor-initiating potential, these transformed FTE-derived SC upon xenotransplantation into immunodeficient mice generated tumors that recapitulated both the histopathological and transcriptomic features of HGSC [162]. Finally, the stemness-related gene *SOX2*, which appears to be causally involved in OC stemness [163], was found to be expressed only in rare FTE cells in women with benign conditions, while *SOX2*-expressing cells were expanded in the FTE of patients affected by HGSC [164]. Moreover, higher frequency of *SOX2*-expressing cells was detected also in the fallopian tubes of women that were at high risk of developing ovarian cancer because of germline BRCA1/2 mutations [164], thus lending further support to the hypothesis that FTE is a prominent site of precursor lesions of HGSC.

### Final remarks on OCSC markers

Based on the studies discussed above, it is striking to see how heterogeneous are the sets of putative OCSC markers used by different groups, especially if compared with other tumor types where there is a more general consensus on the CSC-associated repertoire of surface antigens (e.g., CD44^+^/CD24^-^ in breast carcinoma, LGR5^+^ in colon carcinoma, CD133^+^ in glioblastoma). The variegated picture in OCSC markers is likely due to a combination of different factors. First, the heterogeneity of the disease itself, which encompasses the different histotypes (likely reflecting different tissues of origin and tumor precursors) as well as the molecular alterations, with only a handful of genomic lesions shared by tumors of the same group (e.g. p53 in HGSC) and, otherwise, a very heterogeneous mutational landscape. Second, different sets of OCSC markers might simply reflect the existence of different pools of OCSC. In fact, the heterogeneity of CSC is common across various tumor types [38], which apparently include OC. Indeed, Boesch et al. screened several OC cell lines and found that their SP compartment, while enriched in CSC functional properties, contained different cell subsets with distinct surface markers [146]. It is conceivable, therefore, that cell pools with different phenotypical (and maybe biological) properties share stemness and tumor-propagating abilities. On the other hand, these findings are also compatible with a pronounced plasticity of OCSC, whereby the expression of different markers underlies different phases of the disease or different states of cell differentiation. The two phenomena would not be mutually exclusive, implying that the heterogeneity of OCSC might result from both distinct subsets of cells and cell plasticity.

Furthermore, while one would expect OCSC to represent a minor subpopulation of cancer cells, the prevalence of cells expressing putative CSC markers, such as CD44 and CD24, is often high in OC specimens. On one hand, this might reflect a massive shift of the bulk tumor cell population towards an undifferentiated phenotype (which indeed can be the case in some HGSC and carcinosarcoma). On the other hand, it is highly unlikely that all marker-positive cells identified through these markers are actually *bona fide* CSC. Rather, CSC would probably account only for subsets of that population, which implies the need to employ more stringent marker combinations and/or to associate marker expression with other biological CSC features.

### The stem cell niche: a specialized microenvironment

The acquisition and the maintenance of the biological properties associated with stemness are driven, both in normal and neoplastic tissues, by the interplay between cell-intrinsic characteristics and the interaction with the local microenvironment. Such a microenvironment often consists of a distinct anatomical site within the tumor mass, the so-called stem cell niche. It has become clear that all niche components, i.e. non-stem cancer cells, other host cells, extracellular matrix and soluble factors, regulate various aspects of stem cell biology, including quiescence, mode of division (symmetric versus asymmetric), differentiation, EMT and plasticity. The biological, molecular and functional features of the CSC niche, as well its clinical implications, have been comprehensively discussed in recent reviews [38, 165, 166].

#### The normal SC niche

With regard to the ovary, the nature and the properties of the niche for either normal SC or CSC have remained largely elusive. In the case of normal OSE and FTE, the two major sources of OC, our poor knowledge on somatic SC niches is likely a consequence of the issues and controversies related to the identification of the SC themselves (see section on “The normal counterpart of OCSC”).

##### OSE niche

As reported earlier, Flesken-Nikitin and co-workers identified putative OSE SC in the hilum region of mouse ovary, namely in the junctional area between OSE, mesothelium and fallopian tube [158]. While these observations point to that anatomical site as a plausible SC niche, the components of the niche itself and their regulatory role in OSE SC function remain to be defined. Furthermore, it is unclear to what extent this niching effect of the mouse hilum also extends to the human system, where areas of transition between OSE, mesothelium and tubal epithelium are less defined. It is also noteworthy that other investigators detected somatic SC in the OSE which, however, were widespread throughout the surface rather than restricted to specific areas [151, 159]. Consistent with this pattern, various components of the ovarian cortex have been proposed to have a SC niching function, including mature OSE cells, follicles, follicular fluid, and the stroma underneath the OSE [167].

##### FTE niche

The SC niche in the fallopian tube remains an elusive entity. As reported earlier, a few studies have identified somatic SC in the distal portion of FTE [152, 159–161]. In some cases, the localization of SC at the base of tubal villi [160] pointed to a specialized microenvironment which, however, has not been characterized yet. Based on the physical proximity of the distal fallopian tube with the ovary, it is conceivable that SC in FTE and in OSE share at least some of the niche components and signals [167]. Novel insights into these issues should come from the organoid culture, a methodology that recapitulates SC-driven morphogenesis in vitro [168]. Indeed, the recent application of this technique to primary FTE cells has revealed the major role of the Notch and Wnt signalling pathways as niche factors that regulate stemness and differentiation [169]. Future studies should aim at assessing the relative contributions of other components of the niche (e.g., non-epithelial cells) to the function of normal SC in FTE and OSE.

#### The OCSC niche

##### OCSC niche in solid tumors

The characterization of the niche(s) that supports OCSC must take into account the clinical course of the disease. One can expect that in OC lesions still localized within the ovary and/or the tube, the CSC compartment benefits from the same niche that operates for the normal somatic SC of those tissues (in addition, of course, to the niche factors derived from the tumor itself). On the other hand, the natural evolution of the disease, and especially its peritoneal dissemination, implies the existence of multiple types of niches that support the pathobiological function of OCSC in different anatomical districts. The high rate of OC relapse in peritoneal organs implies that the latter provide a microenvironment which not only protects quiescent disseminated OCSC in the presence of unfavourable conditions (such as during chemotherapy), but also sustains their tumorigenic activity in the context of OC recurrence. These events are very likely controlled by the bidirectional exchange of signalling cues between niche cells and OCSC. For example, organotypic 3D cultures that recapitulated the early dissemination of OC into peritoneal mesothelium revealed that cancer cells, via secretion of transforming growth factor beta-1 (TGFβ1), induce mesothelial cells to synthesize fibronectin [170]. The latter is essential for the adhesion, proliferation and invasion of OC cells and, hence, for metastasis development [170]. More recently, OC-derived exosomes were reported to transfer CD44 into mesothelial cells, resulting in upregulation of matrix metalloproteinase 9 (MMP9) that, in turn, favoured cancer cell homing and invasion. The adipose tissue, especially within the omentum, also provides an optimal environment for the formation of OC lesions. Coculture experiments have shown that omental adipocytes enhance homing, migration and invasion of OC cells and act as an energy source to sustain their metastatic potential [171]. While these studies have not directly addressed the specific impact of microenvironmental factors on OCSC, they highlighted suitable approaches and tools to explore the cellular and molecular players involved in the crosstalk between OCSC and their niche.

##### OCSC niche in ascites

The ascites that develops in advanced OC, both at diagnosis and upon recurrence and development of chemoresistance [172], represents a unique type of tumor microenvironment. Indeed, ascites contains malignant cells which are able to survive and to proliferate even under non-adherent conditions, leading to self-organized spheroids of OC cells that, in turn, account for peritoneal seeding. In addition, ascites contains a wide spectrum of cellular and acellular components that provide a unique microenvironment to malignant OC cells [173]. Several studies have reported that ascites is a rich source of cells with OCSC traits [50–52, 54, 145, 174]. Based on these premises, ascites can be viewed as a specialized OCSC niche, and future research should unravel the ascitic factors that are specifically involved in the regulation of OCSC. For example, interleukin-6 (IL-6) is elevated in ascites [175–179]. IL-6 triggers the JAK/STAT3 signalling pathway [180], which plays an important role in OCSC function [104]. Finally, ascites-derived OC cells display high levels of STAT3 activation [181]. Taken together, these findings suggest that the IL-6/JAK/STAT3 axis is an important effector of the “communication” between OCSC and their niche within the ascites microenvironment. The Wnt signalling pathway likely represents another candidate for such a communication, based on the enrichment for Wnt ligands in OC ascites [182, 183], the activation of Wnt pathway in ascites-derived OCSC [184], and the functional contribution of Wnt signalling to OC stemness [185, 186].

These results have relevant implications also from a clinical standpoint, since drugs targeting STAT3 and Wnt pathways appear particularly effective against ascites-derived malignant OC cells [187, 188].

## Conclusions and future perspectives

In spite of the challenges and limitations related to the intrinsic complexity of OC itself and of its current experimental models, there is no doubt that our knowledge on such an elusive biological entity as OCSC has made tremendous progress over the last few years. The clinical utility of this knowledge, which has started to emerge at least in the context of prognosis and prediction of response to chemotherapy, will hopefully become clearer when translated into novel therapeutic approaches. However, the future research effort towards such a challenging objective will have to take into account, *in primis*, the heterogeneity of OC. One can envision that, in view of OCSC-based precision medicine, it will be necessary to develop appropriate OCSC models that are specific not only for a given OC variant, but even for the individual patient. Along the same line, an appropriate design of clinical trials addressing OCSC-related questions should incorporate the high heterogeneity of the disease with regard to OCSC markers (even the most common markers are found in only <40% tumors), which would impose a careful selection of the right patient cohorts. In addition, monitoring OCSC in the course of clinical trials should entail longitudinal biopsies, while most translational studies in OC focus on the primary tumor. Indeed, OCSC are supposed to account for a very small subpopulation in the primary tumor but should enrich in recurrent disease, both because of their expansion to fuel the relapse and because of the possible selection of drug-resistant OCSC after the first-line treatment.

The successful elimination of OCSC would have tremendous implications for the clinical management of patients. Indeed, it would offer the unprecedented chance of targeting the *driving force* of disease dissemination and recurrence while, at the same time, removing the major cause of tumor resistance to conventional chemotherapy. This makes the combination of classical treatments and OCSC-based therapies a very attractive and promising strategy towards the eradication of OC, with the potential to impact significantly the outcome of OC patients, an objective that we basically failed to accomplish in the last 40 years.
